# Special Issue: Novel Vaccines and Vaccine Technologies for Emerging Infections

**DOI:** 10.3390/vaccines14030206

**Published:** 2026-02-26

**Authors:** Wangxue Chen, Xuguang Li, Amine A. Kamen

**Affiliations:** 1 Human Health and Therapeutics Research Center (HHT), National Research Council Canada, Ottawa, ON K1A 0R6, Canada; 2 Centre for Biologics Evaluation, Biologics and Radiopharmaceutical Drug Directorate, Health Products and Food Branch (HPFB), Health Canada, WHO Collaborating Center for Standardization and Evaluation of Biologicals, Ottawa, ON K1A 0K9, Canada; sean.li@hc-sc.gc.ca; 3 Department of Bioengineering, McGill University, Montreal, QC H2X 1Y4, Canada; amine.kamen@mcgill.ca

Emerging and re-emerging infectious diseases continue to pose a significant threat to global public health, economic prosperity, and societal resilience. Over the recent decades, outbreaks caused by pathogens such as the Ebola virus, the Zika virus, Middle East respiratory syndrome coronavirus (MERS-CoV), and, most recently, SARS-CoV-2, H5N1, and re-emerging monkeypox have underscored the unpredictability of emerging pathogens and the persistent vulnerability of human populations to novel and rapidly evolving infectious agents. Increased globalization and human mobility, urbanization, human–animal interactions, climate change, ecological disruption, and zoonotic spillover have further accelerated the emergence and spread of infectious diseases [1]. Thus, there is a critical need for effective preventive strategies that can be rapidly designed, manufactured, and deployed. In this context, advances in vaccine technologies have become central to strengthening global preparedness and response capabilities.

Vaccination has long been one of the most powerful, successful, and cost-effective tools in preventing infectious diseases and mitigating the impact of epidemics and pandemics [2]. However, most current vaccine development approaches often face limitations and challenges, including long development timelines, manufacturing constraints, and suboptimal efficacy against rapidly mutating pathogens, in addressing rapidly emerging infectious threats. Recent advances in vaccinology, including nucleic acid-based vaccines (mRNA and DNA), viral vector platforms, recombinant protein and nanoparticle vaccines, structure-guided antigen design, and novel adjuvants, have transformed the landscape of vaccine research and development. The unprecedented success of mRNA vaccine development and deployment in response to the COVID-19 pandemic has showcased the potential and feasibility of next-generation vaccine technologies, enabling rapid responses to global public health threats [3].

The tremendous global political enthusiasm, research efforts, and financial investment in vaccine development during and post the COVID-19 pandemic led to significant advances in many aspects of enabling vaccine technologies (such as bioprocessing, cold-chain optimization, and modular manufacturing) and the renewed recognition of the importance of mucosal immunity and vaccination. These advances are likely to accelerate vaccine development, production, and distribution for future responses to pandemics and emerging infections.

This Special Issue aims to provide a platform for sharing the most recent advances in the development of novel enabling vaccine technologies for combating emerging infections and future pandemics. It brings together seven original research papers and three review articles that span the full vaccine development life cycle from antigen discovery and engineering, adjuvant and delivery systems, formulation design and optimization, and manufacturing and analytical innovation. Collectively, these contributions illustrate how technological innovation can be rapidly adapted to accelerate the development of safe, effective, and globally accessible vaccines for current and future emerging infections.

A significant portion of the Special Issue focuses on vaccine formulation stability and cold-chain-independent technologies, reflecting their critical role in real-world vaccine deployment. The Vesicular Stomatitis Virus (VSV) is a clinically relevant viral vector and forms the basis of the Merck Co. licensed Ebola vaccine Ervebo [4]. This vaccine has played an important role in controlling Ebola outbreaks in West Africa. However, its limited thermostability and requirement for −70 °C storage have significantly constrained its deployment. The manuscript by Khan et al. investigates the feasibility of freeze-drying a VSV-based vaccine expressing the SARS-CoV-2 spike protein, focusing on the functional role of excipients, particularly cryoprotectants [5]. Long-term stability data demonstrate that lyophilization enables storage of VSV-vectored vaccines in solid form for six months at 2–8 °C, reducing reliance on ultra-low-temperature cold-chain logistics, improving the potential for equitable global distribution, and contributing knowledge on thermostability critical for viral vector vaccine distribution. The success of Pfizer-BioNTech (Comirnaty) and Moderna (Spikevax) mRNA vaccines during the COVID-19 pandemic represents a major milestone in vaccine biotechnology. However, both vaccines exhibit limited thermostability—Comirnaty requires storage at −80 °C and Spikevax at −20 °C, each with a shelf life of up to six months. These ultra-low-temperature storage requirements impose substantial logistical burdens and restrict global access. Building on insights from the above VSV-vectored vaccine freeze-drying study [5], Khan et al. reviewed emerging literature on the freeze-drying of mRNA–lipid nanoparticle (mRNA-LNP) vaccines [6]. Recent findings provide strong evidence that the field is rapidly advancing, supported by a deeper understanding of biologics formulation and optimized lyophilization protocols involving well-defined formulations and carefully controlled primary and secondary drying steps. This trend is further supported by recent publications [7], which collectively address one of the most significant barriers to global and equitable distribution of mRNA vaccines.

Advances in manufacturing and bioprocessing technologies further illustrate how scalable vaccine production can be enabled through process innovation. The manuscript by Shen et al. builds on a decade of continuous development in media formulation and feeding strategies designed to overcome limitations in high-yield adenovirus vector manufacturing [8]. Adenovirus vectors have demonstrated effectiveness and safety as vaccine platforms during the COVID-19 pandemic, contributing to the vaccination of more than two billion people and saving millions of lives [9]. They also remain the predominant vectors used in many oncotherapy applications [10]. Compared with other viral vectors, adenovirus vector manufacturing is highly advanced and scalable, supported by robust processes that have enabled widespread clinical deployment. The manuscript revisits a critical manufacturing constraint commonly referred to as the cell density effect, a metabolic limitation that restricts production at high cell densities [11]. The authors optimize a fed-batch strategy that supports adenovirus vector production at 5 × 10^6^ cells/mL, achieving titers of 3 × 10^10^ viral particles/mL [8], one of the highest yields reported to date.

Manuscripts by Moon et al. [12] and Cheng et al. [13] on antigen design and presentation technologies highlight how rational epitope selection, structure-guided antigen design, and multivalent display using novel nanoparticle properties can enhance immunogenicity and tailor the desired immune responses. Moon et al. explored a peptide-based vaccine strategy targeting fusion glycoprotein-derived epitopes of the Nipah virus in a murine model [12]. Selected peptides induced antigen-specific humoral and cellular immune responses, demonstrating proof-of-concept that epitope-focused immunization can generate measurable immunity against a high-consequence pathogen. While protective efficacy was partial and remains exploratory, the study supports peptide vaccines as a modular and potentially safer platform for emerging and biosafety level-4 pathogens, particularly for early-stage development and rapid-response scenarios. The manuscript by Cheng et al. reports the construction of a versatile, single-component, self-assembling nanoparticle vaccine for SARS-CoV-2 by genetic fusion of the spike protein to a bacterial lumazine synthase (LuS) nanoparticle platform [13]. The prefusion-stabilized S proteins displayed by LuS nanoparticles preserved native antigenicity and elicited significantly higher anti-RBD IgG and potent neutralizing antibody titers in mice at very low antigen doses (0.08 µg) as compared to the soluble spike trimers. Heterologous boosting with a variant LuS nanoparticle ~38 weeks after priming resulted in 25- to 126-fold increases in spike protein-binding antibody titers above pre-boost levels (exceeding initial peak responses). Cross-neutralizing activity against SARS-CoV-1 was also observed in some animals. Importantly, combined intramuscular DNA prime and intranasal LuS nanoparticle boosting induced mucosal IgA responses in respiratory secretions. These results demonstrate the potential of this platform to induce both systemic and mucosal immunity of long duration and wide breadth.

Vaccine adjuvants are immunostimulatory agents that play crucial roles in enhancing vaccine safety and efficacy. They are particularly important for improving immune responses in elderly or immunocompromised populations, enabling antigen dose sparing, and reducing the number of boosters required [14]. These features are especially vital for pandemic preparedness, where rapid, broad, and durable protection is needed across diverse populations. The manuscript by Stark et al. evaluated the immunogenicity of SARS-CoV-2 spike protein booster vaccines formulated with or without a sulfated lactosyl archaeol (SLA) archaeosome adjuvant in young and aged mice that had been previously primed with an ancestral (original) strain spike vaccine [15]. All booster formulations (ancestral, beta, and delta spike antigens) enhanced humoral immune responses over pre-boost levels, but the immune imprinting from the original priming series limited neutralization antibody responses against the corresponding variants. However, the inclusion of SLA archaeosome adjuvant consistently enhanced antigen-specific T-cell responses compared to non-adjuvanted boosters. This was especially notable in aged mice, where immune responses are generally weaker. This study highlights that adjuvant and formulation selection can be as important as the antigen sequence in designing effective booster vaccines and underscores the potential benefits of SLA archaeosomes in immunocompromised populations.

The manuscript by Caldarelli et al. provides a systematic overview of the current understanding of ASIA (Autoimmune/Inflammatory Syndrome Induced by Adjuvants) syndrome [16]. ASIA is a proposed conceptual framework describing clusters of clinical reactions temporally associated with immune stimulation, such as vaccination, silicone exposure, or other adjuvants; however, causality has not been established. Reported clinical manifestations are highly heterogeneous and non-specific, influenced by multiple variables such as genetic background, age, sex, and underlying medical conditions. Importantly, ASIA lacks validated biomarkers and remains largely transient and observational in nature. Large-scale pharmacovigilance and epidemiological studies fail to demonstrate an increased incidence of vaccine-associated autoimmune disease at the population level. While rare susceptible individuals may exist, this does not translate into a public health risk, and population-level data overwhelmingly support vaccine safety. As such, ASIA should be viewed as a hypothesis-generating concept that warrants further investigation, particularly through the identification of susceptibility markers, systems immunology approaches, and longitudinal immune monitoring. Collectively, these papers [15,16] illustrate how formulation and adjuvant technologies can modulate immune quality, particularly in populations with altered immune responsiveness. At the same time, broader discussions of adjuvant-associated immune activation underscore the need for balanced immune enhancement and continued mechanistic understanding of adjuvant–host interactions.

The Special Issue also highlights the growing importance of analytical and computational technologies in vaccine development. The manuscript by Lorbetskie et al. applied machine learning approaches to reversed-phase chromatography data to improve analytical characterization of influenza vaccines [17]. By analyzing a wide range of antigens and formulations, the study demonstrates that machine learning can enhance the resolution, consistency, and interpretability of complex chromatographic profiles, allowing better discrimination of antigen variants, degradation products, and formulation differences. This work addresses a key challenge in vaccine manufacturing and quality control, highlighting how advanced analytics can support batch comparability, regulatory oversight, and scalable vaccine production without altering the immunological components themselves.

In parallel, studies by Ferraresi et al. examined why immune responses to hepatitis B vaccination decline with age, focusing on epigenetic mechanisms [18]. The authors demonstrate that vaccine responsiveness is linked to age-related epigenetic drift, notably DNA methylation changes affecting genes responsible for antigen presentation and adaptive immune activation. Individuals with weaker antibody responses exhibited distinct epigenetic signatures, suggesting that vaccine hyporesponsiveness reflects not only immunosenescence but also stable regulatory changes in immune cells. These findings support the concept of precision vaccinology and suggest that epigenetic markers may help predict vaccine efficacy or guide tailored vaccination strategies.

Finally, the manuscript by Perera et al. provides a comprehensive review of the mechanisms and applications of high-energy electron beam (eBeam) irradiation as an alternative method for producing inactivated vaccines against bacterial, viral, and protozoan pathogens [19]. The authors detail how eBeam causes extensive nucleic acid damage that fully inactivates microorganisms while largely preserving surface antigen structures to maintain immunogenic epitopes. eBeam has successfully inactivated a wide range of pathogens, including *Salmonella*, *Clostridium perfringens*, influenza A virus, and respiratory syncytial virus, and the resulting vaccines elicited protective immune responses in animal models. The review also highlights the practical advantages of eBeam technology over many chemical or heat inactivation methods, including better antigen integrity, using less hazardous chemicals, rapid processing times, and cost-effectiveness. This positions eBeam technology as a promising tool for the development of safer and more effective inactivated vaccines compared to traditional approaches.

Taken together, the 10 contributions presented in this Special Issue collectively highlight the transformative impact of novel enabling vaccine technologies that can be broadly applied, rapidly adapted, and efficiently scaled in response to future emerging infectious threats. These innovative technologies are redefining how modern vaccinology is evolving to meet the demands of an increasingly complex and unpredictable infectious disease landscape. Importantly, they also illustrate how lessons learned from recent outbreaks, particularly the COVID-19 pandemic, are shaping more agile, scalable, and responsive vaccine development pipelines. Such technology-driven approaches will be essential as the pace of pathogen emergence continues to accelerate.

Despite remarkable progress, the work presented in this Special Issue also highlights ongoing challenges that must be addressed to fully realize the potential of next-generation vaccine technologies. These include improving the breadth and durability of vaccine-induced immune responses, enhancing thermostability and manufacturability, ensuring regulatory readiness for novel approaches, and achieving equitable global access. To address these challenges, sustained investment in basic research (immunology, computational design, and systems vaccinology), surveillance, bioengineering, data science, manufacturing, and pandemic preparedness will be critically needed. Moreover, close collaboration among academia, industry, regulatory bodies, and public health organizations is essential.

We hope that this collection will inform future research efforts, stimulate continued innovation and collaboration, and support the development of next-generation vaccines capable of preventing future outbreaks and enhancing global health security. As emerging infections continue to pose unpredictable risks, proactive and innovative vaccine strategies will remain central to pandemic preparedness and global health security.
